# Impact of the COVID-19 pandemic on the incidence and clinical outcomes of diabetic ketoacidosis among male and female children with type 1 diabetes: systematic review and meta-analysis

**DOI:** 10.12688/f1000research.128687.2

**Published:** 2023-08-10

**Authors:** Edinson Dante Meregildo-Rodriguez, Franco Ernesto León-Jiménez, Brenda Aurora Dolores Tafur-Hoyos, Gustavo Adolfo Vásquez-Tirado

**Affiliations:** 1 Escuela de Medicina, Universidad César Vallejo, Trujillo, La Libertad, Peru; 2 Hospital Regional Lambayeque, Chiclayo, Lambayeque, Peru; 3 Escuela de Medicina, Universidad Privada Antenor Orrego, Trujillo, La Libertad, Peru

**Keywords:** COVID-19, SARS-CoV-2, type 1 diabetes mellitus, diabetic ketoacidosis, pediatrics, child, systematic review, meta-analysis

## Abstract

**Background:** Some studies suggest that the SARS-CoV-2 pandemic increased the incidence of type 1 diabetes mellitus (T1DM) and diabetic ketoacidosis (DKA). However, the impact of this pandemic on pediatric T1DM is still mostly unknown. Therefore, we aimed to assess the effect of the COVID-19 pandemic on clinical outcomes in children with T1DM.

**Methods:** We systematically searched for six databases up to 31 August 2022. We included 46 observational studies, 159,505 children of both sexes with T1DM, and 17,547 DKA events.

**Results:** The COVID-19 pandemic significantly increased, in both sexes, the incidence of 1) DKA (OR 1.68; 95% CI 1.44–1.96), 2) severe DKA (OR 1.84; 95% CI 1.59–2.12), 3) DKA in newly diagnosed T1DM (OR 1.75; 95% CI 1.51–2.03), and 4) ICU admissions (OR 1.90; 95% CI 1.60–2.26). However, we did not find a significant association between this pandemic and 1) the incidence of T1DM, 2) the incidence of DKA in established T1DM, 3) the incidence of KDA complications, 4) the length of hospitalization stay, and 5) mortality. Subgroup analysis showed that the study design and the continent of origin accounted for the heterogeneity.

**Conclusions:** The pandemic SARS-CoV-2 raised, in both sexes, the risk of DKA, severe DKA, DKA *de novo*, and ICU admissions.

## Introduction

Type 1 diabetes (T1DM) is an autoimmune disease traditionally associated with viral infections. ^1^^,^^2^ The severe acute respiratory syndrome coronavirus 2 (SARS-CoV-2) virus shows a great affinity for the angiotensin-converting enzyme (ACE) 2 receptor and other receptors present in the islets of Langerhans in the pancreas. Therefore, the SARS-CoV-2 virus could induce insulin resistance, hyperglycemia, and diabetes mellitus (DM) decompensation. On the contrary, hyperglycemia could worsen the prognosis of the COVID-19 disease. ^3^^,^^4^ There seems to be, in fact, a bidirectional relation between COVID-19 and DM. ^5^^,^^6^

Some studies suggest that during the COVID-19 pandemic, the risk of T1DM has increased. ^7^^–^^9^ Similarly, three systematic reviews and meta-analyses have shown an increase in the risk of developing DKA and severe DKA during the COVID-19 pandemic compared to the pre-pandemic era. ^10^^–^^12^ Besides, people with DM are disproportionately affected by COVID-19. For example, they are more susceptible to be admitted to an intensive care unit (ICU) than those non-diabetic patients. ^13^ In addition, patients with T1DM have 3.5 times higher mortality rates from COVID-19 than those without T1DM. ^14^ However, data are still controversial. Some studies found no differences in the percentage of newly diagnosed T1DM complicating with DKA in COVID-19 and non-COVID-19 periods. ^15^^,^^16^ Therefore, it is maybe due to more difficult access to healthcare systems. ^15^ In fact, despite the advantages of telemedicine during the pandemic, several reports have shown that a considerable quantity of T1DM patients has presented complications, probably associated with fear and delay in seeking medical help. ^17^ Another factor that could increase the incidence of T1DM, diabetic ketoacidosis (DKA), and severe DKA is the widespread use of steroids during the pandemic due to their ability to induce insulin resistance, hyperglycemia, and *de novo* DM. ^18^^,^^19^

An international multicenter study in Europe and the USA aimed to examine the impact of the COVID-19 pandemic on the prevalence of DKA in pediatric type 1 diabetes. The researchers noted that the DKA prevalence at T1DM diagnosis during the pandemic years was 39%, significantly higher than the estimated prevalence of 33% for the two previous years. However, they did not find significant differences by sex or age. ^20^

Although the evidence suggests that the SARS-CoV-2 pandemic has raised the incidence of T1DM, DKA, and severe DKA, information about the impact of COVID-19 on other clinical outcomes is scarce. Thus, we aimed to determine the impact of this pandemic on the probability of developing pediatric T1DM, DKA, severe DKA, DKA in newly diagnosed and established T1DM, ICU admissions, DKA complications, length of hospitalization stay, and mortality due to DKA.

## Methods

We conducted this systematic review and meta-analysis following the recommendations of the Cochrane Handbook, ^21^ the PRISMA, ^22^ and the AMSTAR 2 ^23^ guidelines. We previously registered the protocol in PROSPERO (CRD42021278821). We searched for observational (cohort, case-control, and cross-sectional) studies and randomized control trials published until 31 August 2022, in Medline (PubMed), Google Scholar, Scopus, ScienceDirect, EMBASE, and Web of Science. We combined different keywords, controlled vocabulary terms ( *e.g.*, MeSH and Emtree), and free terms following a predefined PECO framework (population: “children with type 1 diabetes mellitus” OR “children without type 1 diabetes mellitus”; exposure: “COVID-19” OR “SARS-CoV-2”; comparator: “NOT COVID-19” OR “NOT SARS-CoV-2”; outcome: “diabetic ketoacidosis” OR “DKA” OR “incidence” OR “hospital stay” OR “intensive care unit admission” OR “mortality” ( *Extended data*). We did not limit searches by date or language.

The eligibility criteria followed the PECO question. We included studies that evaluated pediatrics’new-onset T1DM during the COVID-19 pandemic and during the same pre-pandemic period, which reported at least one of the following outcomes: children with new-onset and established T1DM, DKA among children with newly diagnosed and established T1DM, DKA, and severe DKA among newly diagnosed and established children with T1DM, ICU admissions, DKA complications, length of hospitalization stay, and mortality due to DKA. We excluded case reports, case series, duplicated publications, and papers in which more than 50% of patients were >18 years old or had other types of diabetes mellitus. This cutoff ensures that most participants in the included studies are children, which increases the likelihood that the results are relevant to them. ^21^ Three reviewers (FEL-J, BADT-H, and GAV-T) independently. Three independent reviewers (FEL-J, BADT-H, and GAV-T) examined articles, and a fourth researcher (EDM-R) resolved discrepancies. We screened references from retrieved documents for additional articles. We reviewed the papers found and verified the compliance of the components of the PECO framework and the inclusion and exclusion criteria. In addition, we extracted and recorded the essential information from each article in a spreadsheet: authors' names, year and country of publication, type of study, number of patients, sex of the patients, number of events, the measure of association, and adjusted confounders if reported.

Initially, we planned to perform subgroup analyses according to DKA severity, mortality, length of hospitalization, and sex. However, as the protocol stipulated, these subgroup analyses would be executed if feasible regarding information and data. In addition, in concordance with the SAGER guidelines, ^24^ we defined sex as the biological attributes associated with physical and physiological features separated as mutually exclusive and complementary categories (male or female); that is, the sum of both is equal to the total number of cases.

We pooled the number of patients and events of interest in the quantitative synthesis and calculated odds ratios (ORs) with 95% confidence intervals (95% CIs) using the Mantel-Haenszel method. We considered the risk ratio (RR) equivalent to OR if the incidence of the event evaluated was fewer than 10 percent. ^25^ We used forest plots to represent the quantitative synthesis and assessed heterogeneity among studies with Cochran’s Q test and Higgins I ^2^ statistic. We predefined that if heterogeneity was not significant ( *p* > 0.05, I ^2^ statistics < 40%), we would use a fixed-effects model. We carried out sensitivity and subgroup analyses and assessed the risk of bias with the Newcastle–Ottawa Scale (NOS) tool. ^26^ Finally, we examined the publication bias using a funnel plot.

In addition, we compare the incidence per 10 ^5^ children/year of T1DM between the pre-pandemic and pandemic period using medians and interquartile ranges (IQRs) and the Mann–Whitney test. We calculated the mean difference (MD) measures as the absolute difference between the means in the two (pre-pandemic and pandemic) groups. In the case of those studies that did not report the means or the standard deviation (SD) of the samples, we estimated it from the data provided by the authors. As long as the data are not significantly skewed away from normality, ^27^ it is possible to estimate MD and SD if we know the size, the minimum, median, and maximum of the sample (scenario 1); ^28^^,^^29^ the size, first quartile, median, third quartile (scenario 2); ^28^^,^^29^ or the size, minimum, first quartile, median, third quartile, and maximum of the sample (scenario 3). ^28^^,^^30^

## Results

**Figure 1.**
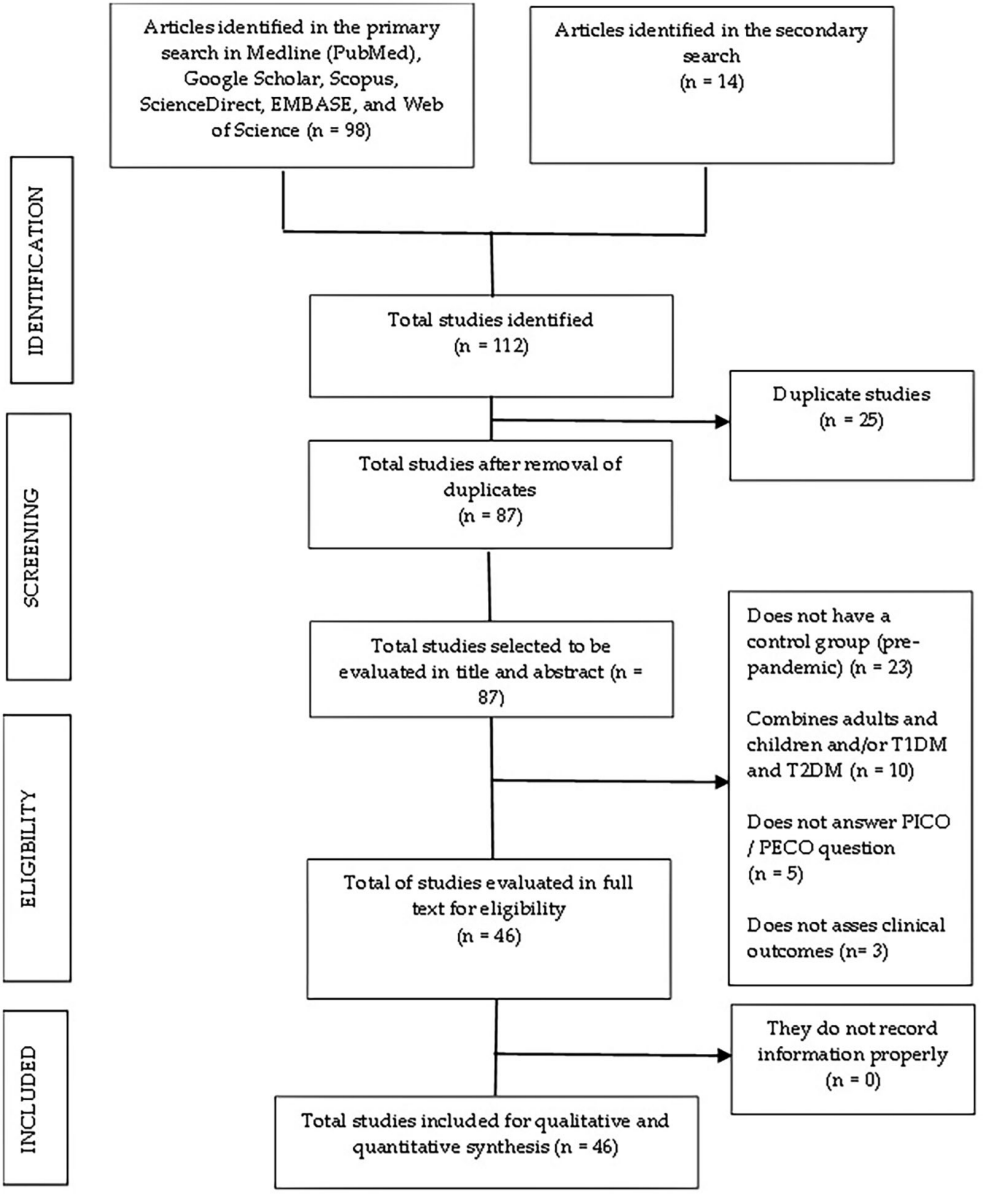
Flow chart of the selection process of the included studies.

We collected 112 studies, 98 in the primary screening and 14 in the secondary examination. Following the removal of duplicated articles, there were 87 articles left that we examined in title and abstract. Subsequently, we found and analyzed 46 papers in full text. We considered these 46 papers for qualitative and quantitative synthesis ( Figure 1).

Of the 46 studies included in this review, six studies were cross-sectional studies (CSS), two papers were case-control studies (CCS), and thirty-eight documents were prospective or retrospective cohort studies (PCS, RCS). This review includes a total of 159,505 children with T1DM, 17,547 events of DKA—5,792 episodes of severe DKA, 15,600 episodes of DKA in *de novo* T1DM, and 521 episodes of DKA in established T1DM, 791 ICU admissions, 822 DKA related complications, and one death ( Table 1).

**Table 1.** General characteristics of the included studies.

Study	Age (years)	DKA criteria	Group	Male sex (%)	Total (N)	DKA	Severe DKA	De novo T1DM	Established T1DM	ICU	Complications	Hospital stay	Dead
Kamrath C. ^17^ 2020. (MC) PCS. Germany.	≤ 18	Other	Pre-COVID	517 (53.9)	959	233	126	233	ND	ND	ND	ND	ND
			COVID	327 (61.5)	532	238	103	238	ND	ND	ND	ND	ND
Al-Abdulrazzaq D. ^31^ 2021. (MC) PCS. Kuwait.	≤ 12	ISPAD	Pre-COVID	150 (49.5)	303	113	33	113	ND	33	ND	ND	ND
			COVID	151 (46.6)	324	166	60	166	ND	64	ND	ND	ND
Alaqeel A. ^32^ 2021. (MC) RCS. Saudi Arabia.	1–14	ADA	Pre-COVID	69 (44.8)	154	112	24	15	97	ND	ND	2.9 ± 0.1	ND
			COVID	51 (48.1)	106	88	23	23	65	ND	ND	2.9 ± 0.2	ND
Alassaf A. ^33^ 2022. (UC) RCS. Jordan.	ND	ISPAD	Pre-COVID	37 (44.6)	83	29	8	29	ND	ND	ND	ND	ND
			COVID	28 (51.9)	54	28	9	28	ND	ND	ND	ND	ND
Atlas G. ^34^ 2020. (MC) RCS. Australia.	ND	Other	Pre-COVID	118 (57.8)	204	86	32	86	ND	25	ND	ND	ND
			COVID	32 (55.2)	58	30	13	30	ND	15	ND	ND	ND
Boboc AA. ^35^ 2021. (UC) RCS. Romania.	< 18	ISPAD	Pre-COVID	170 (54.5)	312	123	33	123	ND	ND	ND	ND	ND
			COVID	75 (51.0)	147	97	41	97	ND	ND	ND	ND	ND
Bogale KT. ^36^ 2021. (UC) RCS. USA.	≤ 18	Other	Pre-COVID	218 (58.9)	370	172	123	172	ND	ND	20	ND	ND
			COVID	23 (54.8)	42	20	13	20	ND	ND	2	ND	ND
Chambers MA. ^37^ 2022. (UC) RCS. USA.	< 18	ISPAD	Pre-COVID	167 (52.2)	320	175	58	175	ND	157	ND	2.98 ± 0.17	0
			COVID	83 (54.6)	152	98	59	98	ND	116	ND	3.03 ± 0.18	0
Cherubini V. ^38^ 2022. (MC) RCS. Italy.	≤ 18	Other	Pre-COVID	ND	3068	1071	319	1071	ND	ND	ND	ND	ND
			COVID	ND	1169	460	166	460	ND	ND	ND	ND	ND
Danne T. ^39^ 2021. (MC) CCS. Germany.	≤ 21	ISPAD	Pre-COVID	16254 (52.0)	31258	280	ND	ND	ND	ND	ND	ND	ND
			COVID	13178 (51.6)	25543	228	ND	ND	ND	ND	ND	ND	ND
Dilek SÖ. ^40^ 2021. (UC) CSS. Turkey.	< 18	ISPAD	Pre-COVID	21 (45.7)	46	27	4	27	ND	ND	7	ND	ND
			COVID	35 (47.3)	74	68	15	68	ND	ND	17	ND	ND
Donbaloğlu Z. ^41^ 2020. (UC) CSS. Turkey.	< 18	ISPAD	Pre-COVID	ND	78	43	20	43	ND	ND	ND	ND	ND
			COVID	29 (51.8)	56	30	13	30	ND	ND	ND	ND	ND
Dżygało K. ^42^ 2020. (UC) RCS. Poland.	≤ 18	WHO	Pre-COVID	26 (50.0)	52	29	6	29	ND	ND	ND	ND	ND
			COVID	22 (64.7)	34	18	11	18	ND	ND	ND	ND	ND
Fathi A. ^43^ 2022. (UC) RCS. USA.	ND	ND	Pre-COVID	21 (42.0)	50	ND	ND	ND	ND	ND	5	0.76 ± 0.07	0
			COVID	16 (37.2)	43	ND	ND	ND	ND	ND	3	1.02 ± 0.14	1
Goldman S. ^44^ 2022. (MC) RCS. Israel.	< 18	ISPAD	Pre-COVID	181 (49.7)	364	150	38	150	ND	71	ND	3.02 ± 0.51	ND
			COVID	87 (59.6)	146	85	29	85	ND	50	ND	4.00 ± 0.38	ND
Gottesman BL. ^45^ 2022. (UC) CSS. USA.	< 19	ND	Pre-COVID	ND	641	261	ND	261	ND	41	ND	ND	ND
			COVID	81 (43.3)	187	93	ND	93	ND	16	ND	ND	ND
Han MJ. ^46^ 2021. (MC) RCS. South Korea.	≤ 18	Other	Pre-COVID	11 (91.7)	12	8	ND	8	4	ND	10	ND	ND
			COVID	1 (14.3)	7	7	ND	7	0	ND	6	ND	ND
Hawkes CP. ^47^ 2021. (UC) RCS. USA.	< 18	ADA	Pre-COVID	ND	93	35	11	35	ND	ND	ND	ND	ND
			COVID	ND	73	33	11	33	ND	ND	ND	ND	ND
Hernández HM. ^48^ 2022. (MC) RCS. Spain.	< 1	ISPAD	Pre-COVID	27 (51.9)	52	22	ND	22	ND	ND	ND	ND	ND
			COVID	20 (54.1)	37	12	ND	12	ND	ND	ND	ND	ND
Ho J. ^49^ 2021. (MC) RCS. Canada.	< 18	DCCP	Pre-COVID	47 (41.2)	114	52	15	52	ND	9	3	ND	ND
			COVID	46 (43.0)	107	73	29	73	ND	19	13	ND	ND
Jacob R. ^50^ 2021. (MC) CSS. Israel.	≤ 18	ADA	Pre-COVID	ND	154	62	20	31	31	35	ND	ND	ND
			COVID	ND	150	84	26	46	38	40	ND	ND	ND
Kamrath C. ^51^ 2021. (MC) PCS. Germany.	≤ 18	Other	Pre-COVID	ND	42417	7312	2101	7312	ND	ND	ND	ND	ND
			COVID	1799 (55.6)	3238	1094	401	1094	ND	ND	ND	ND	ND
Kaya G. ^52^ 2022. (UC) RCS. Turkey.	<18	ISPAD	Pre-COVID	42 (53.2)	79	32	12	32	ND	ND	ND	15.02 ± 5.53	ND
			COVID	24 (54.5)	44	30	14	30	ND	ND	ND	10.02 ± 3.89	ND
Kiral E. ^53^ 2022. (MC) RCS. Turkey.	< 18	ISPAD	Pre-COVID	241 (46.6)	517	517	292	165	127	ND	378	ND	ND
			COVID	207 (43.1)	480	480	337	226	111	ND	329	ND	ND
Kostopoulou E. ^54^ 2021. (MC) PCS. Greece.	< 18	ND	Pre-COVID	12 (70.6)	17	17	3	6	ND	1	ND	ND	ND
			COVID	9 (42.9)	21	18	14	14	ND	6	ND	ND	ND
Lavik AR. ^55^ 2022. (MC) RCS. USA.	≤ 19	ISPAD	Pre-COVID	ND	1041	547	ND	ND	ND	ND	ND	ND	ND
			COVID	ND	1035	556	ND	ND	ND	ND	ND	ND	ND
Lawrence C. ^56^ 2021. (UC) RCS. Australia.	< 18	ISPAD	Pre-COVID	21 (50.0)	42	11	2	11	ND	ND	ND	ND	ND
			COVID	3 (27.3)	11	8	5	8	ND	ND	ND	ND	ND
Lee MS. ^57^ 2022. (UC) CSS. South Korea.	< 19	ISPAD	Pre-COVID	1 (10.0)	10	4	0	4	ND	ND	ND	ND	ND
			COVID	6 (60.0)	10	6	1	6	ND	ND	ND	ND	ND
Lee Y. ^58^ 2022. (MC) RCS. South Korea.	< 18	ISPAD	Pre-COVID	51 (51.5)	41	16	9	16	ND	ND	3	ND	ND
			COVID	46 (54.8)	51	31	9	31	ND	ND	6	ND	ND
Loh C. ^59^ 2021. (UC) CCS. Germany.	≤ 18	ISPAD	Pre-COVID	36 (49.3)	73	15	6	9	6	ND	ND	9.86 ± 11.02	ND
			COVID	21 (40.4)	52	15	8	8	7	ND	ND	10.13 ± 0.67	ND
Luciano TM. ^60^ 2022. (UC) PCS. Brazil.	< 18	Other	Pre-COVID	14 (56.0)	25	9	3	9	ND	ND	9	ND	ND
			COVID	9 (50.0)	18	12	6	12	ND	ND	11	ND	ND
Mameli C. ^61^ 2021. (MC) PCS. Italy.	< 18	ISPAD	Pre-COVID	293 (47.0)	624	184	62	184	ND	25	ND	ND	ND
			COVID	110 (43.0)	256	91	39	91	ND	17	ND	ND	ND
Marks BE. ^62^ 2021. (UC) RCS. USA.	≤ 21	ADA	Pre-COVID	163 (52.6)	310	145	52	145	ND	ND	ND	ND	ND
			COVID	101 (55.5)	182	105	51	105	ND	ND	ND	ND	ND
Mastromauro C. ^63^ 2022. (UC) RCS. Italy.	≤ 19	ISPAD	Pre-COVID	81 (61.4)	132	48	11	48	ND	ND	ND	ND	ND
			COVID	20 (50.0)	40	22	9	22	ND	ND	ND	ND	ND
McGlacken BSM. ^64^ 2021. (MC) CSS. UK.	< 18	ISPAD	Pre-COVID	15 (50.0)	30	9	3	9	ND	2	ND	ND	ND
			COVID	9 (52.9)	17	13	8	13	ND	4	ND	ND	ND
Mönkemöller K. ^65^ 2021. (MC) PCS. Germany.	< 18	Other	Pre-COVID	223 (23.2)	959	231	125	ND	ND	ND	ND	ND	ND
			COVID	233 (43.8)	532	237	100	ND	ND	ND	ND	ND	ND
Nóvoa MY. ^66^ 2022. (UC) RCS. Spain.	< 14	ADA	Pre-COVID	ND	28	12	ND	12	ND	ND	ND	ND	ND
			COVID	ND	42	19	ND	19	ND	ND	ND	ND	ND
Passanisi S. ^67^ 2022. (MC) RCS. Italy.	≤14	Other	Pre-COVID	18 (41.9)	43	19	8	19	ND	ND	ND	ND	ND
			COVID	51 (45.9)	111	52	20	52	ND	ND	ND	ND	ND
Rabbone I. ^68^ 2020. (MC) PCS. Italy.	< 15	ISPAD	Pre-COVID	ND	208	86	31	86	22	ND	ND	ND	ND
			COVID	ND	160	61	27	61	13	ND	ND	ND	ND
Salmi H. ^69^ 2021. (MC) RCS. Finland.	≤ 15	ND	Pre-COVID	128 (55.4)	231	20	20	20	ND	25	ND	ND	ND
			COVID	48 (57.1)	84	13	13	13	ND	20	ND	ND	ND
Sellers EAC. ^70^ 2021. (MC) RCS. Canada.	ND	ND	Pre-COVID	ND	236	86	39	86	ND	ND	ND	ND	ND
			COVID	ND	260	143	69	143	ND	ND	ND	ND	ND
Tittel SR. ^71^ 2020. (MC) RCS. Germany.	< 18	ND	Pre-COVID	ND	ND	ND	ND	ND	ND	ND	ND	ND	ND
			COVID	ND	532	ND	ND	ND	ND	ND	ND	ND	ND
Vlad A. ^72^ 2021. (MC) RCS.Romania.	< 14	ND	Pre-COVID	ND	ND	ND	ND	ND	ND	ND	ND	ND	ND
			COVID	ND	ND	ND	ND	ND	ND	ND	ND	ND	ND
Vorgučin I. ^73^ 2022. (MC) RCS. Serbia.	< 19	ISPAD	Pre-COVID	73 (55.3)	132	45	16	45	ND	ND	ND	ND	ND
			COVID	50 (50.5)	99	42	15	42	ND	ND	ND	ND	ND
Wolf RM. ^74^ 2022. (MC) RCS. USA.	≤ 26	ISPAD	Pre-COVID	622 (48.7)	17749	493	145	493	ND	ND	ND	ND	ND
			COVID	648 (46.3)	17597	599	215	599	ND	ND	ND	ND	ND
Zubkiewicz A. ^75^ 2021. (MC) RCS. Poland.	< 18	ISPAD	Pre-COVID	1054 (53.7)	1961	ND	ND	ND	ND	ND	ND	ND	ND
			COVID	ND	ND	ND	ND	ND	ND	ND	ND	ND	ND

T1DM: type 1 diabetes mellitus, DKA: diabetic ketoacidosis, RCS: Retrospective cohort study, PCS: Prospective cohort study, CSS: Cross-sectional study, ISPAD: International Society for Pediatric and Adolescent Diabetes, ADA: American Diabetes Association, DCCP: Diabetes Canada Clinical Practice, WHO: World Health Organization, ICU: admission to the intensive care unit, UC: unicenter, MC: multicenter, ND: not described.

Following the approach of most studies, we analyzed outcomes comparing the pre-pandemic and the pandemic periods—regardless of their COVID-19 status (positive or negative)—instead of reporting events in children with COVID-19 positive or negative. Consequently, we only included papers that reported both groups of children (a pre-pandemic and a pandemic cohort). The lack of a pre-pandemic group was the leading cause of the exclusion of most studies ( ***Extended data***).

We analyzed nine outcomes (pre and during the COVID-19 pandemic) in children: 1) the incidence of T1DM, 2) the incidence of DKA in T1DM, 3) the incidence of severe DKA in T1DM, 4) the incidence of DKA in *de novo* T1DM, 5) the incidence of DKA in established T1DM, 6) the incidence of ICU admissions due to DKA in T1DM, 7) the incidence of DKA complications in T1DM, 8) the length of hospitalization stay, and 9) the risk of mortality due to DKA.

T1DM: type 1 diabetes mellitus, DKA: diabetic ketoacidosis, RCS: Retrospective cohort study, PCS: Prospective cohort study, CSS: Cross-sectional study, ISPAD: International Society for Pediatric and Adolescent Diabetes, ADA: American Diabetes Association, DCCP: Diabetes Canada Clinical Practice, WHO: World Health Organization, ICU: admission to the intensive care unit, UC: unicenter, MC: multicenter, ND: not described.

### Incidence of T1DM

During the pre-COVID-19 era, the median incidence of DKA among children with T1DM was 17.28 per 10 ^5^ patients/year (IQR 10.87–26.9), and during the COVID-19 era, the incidence of DKA among children with T1DM was 19.35 per 10 ^5^ patients/year (IQR 14.65–34.7). However, this difference was not statistically significantly ( *p* = 0.41, Mann–Whitney test).

### Incidence of DKA among T1DM pediatric patients

**Figure 2.**
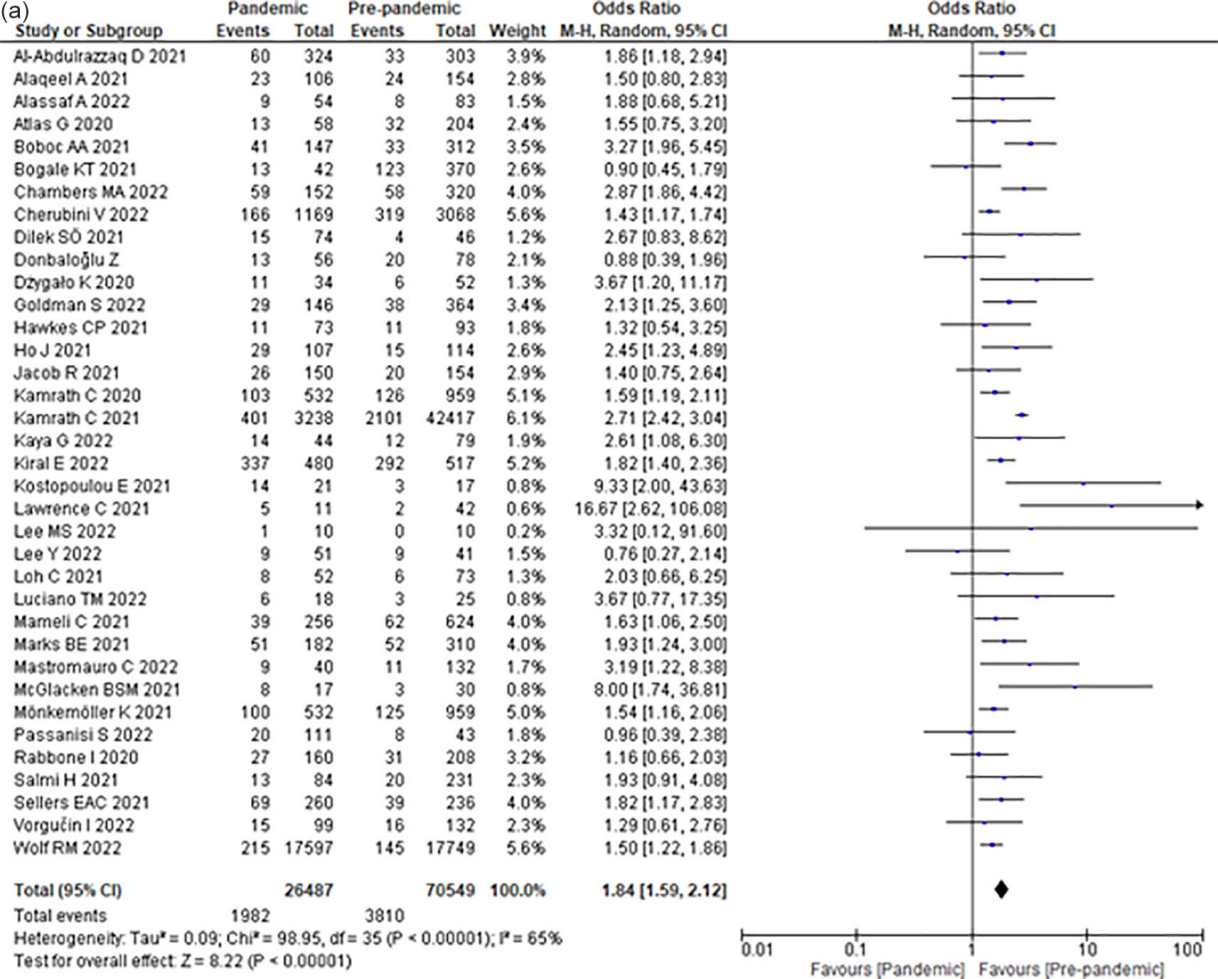

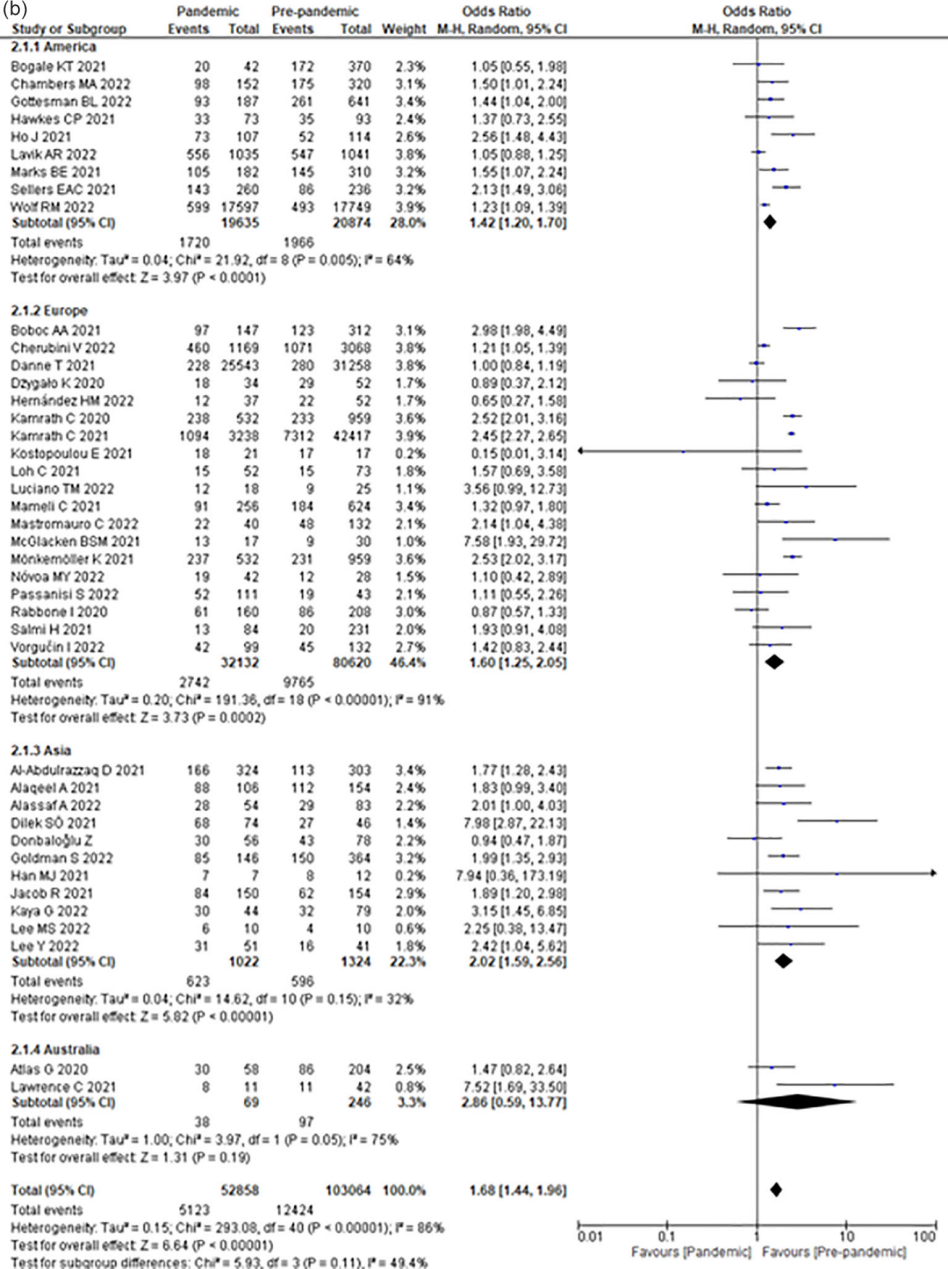

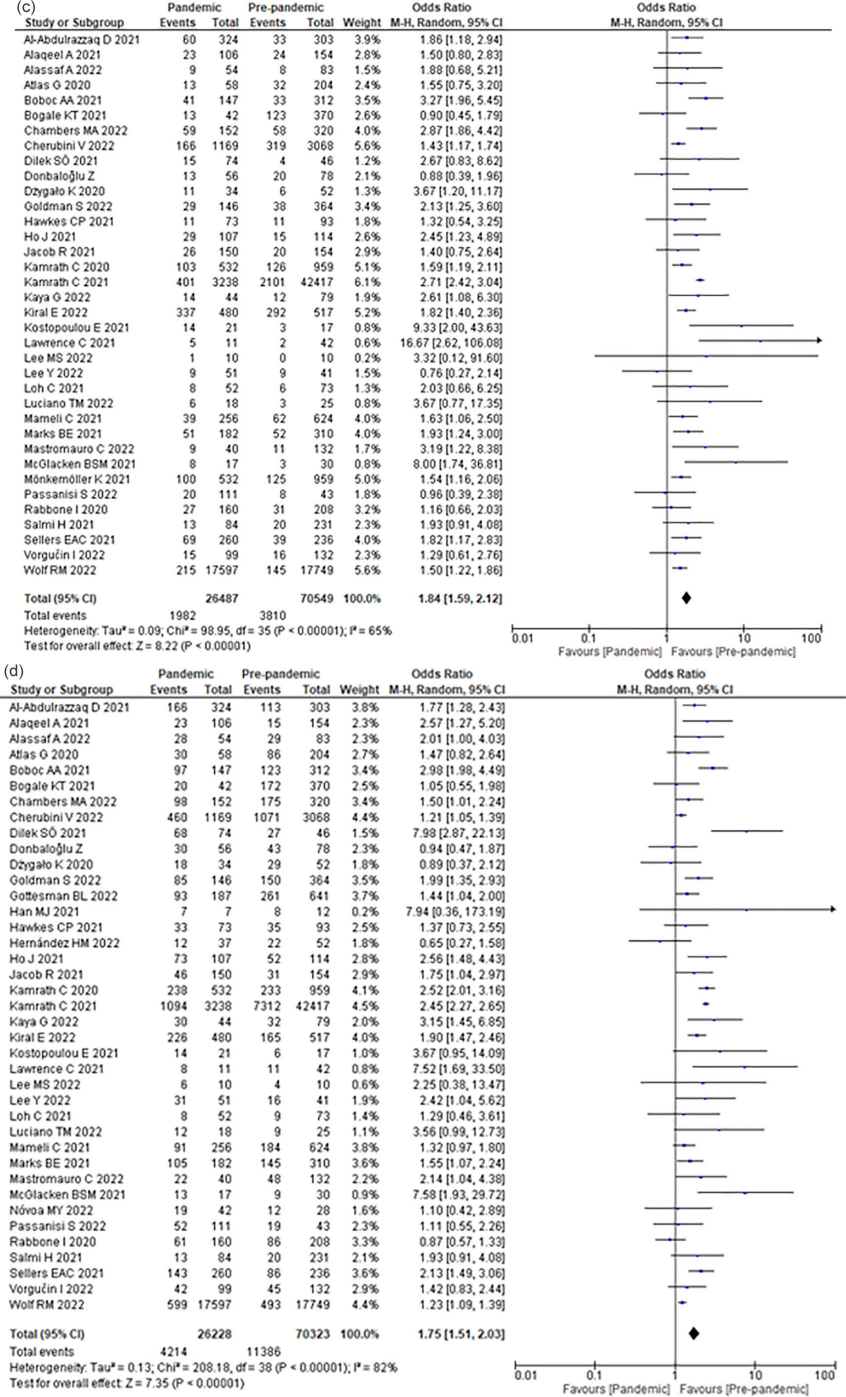

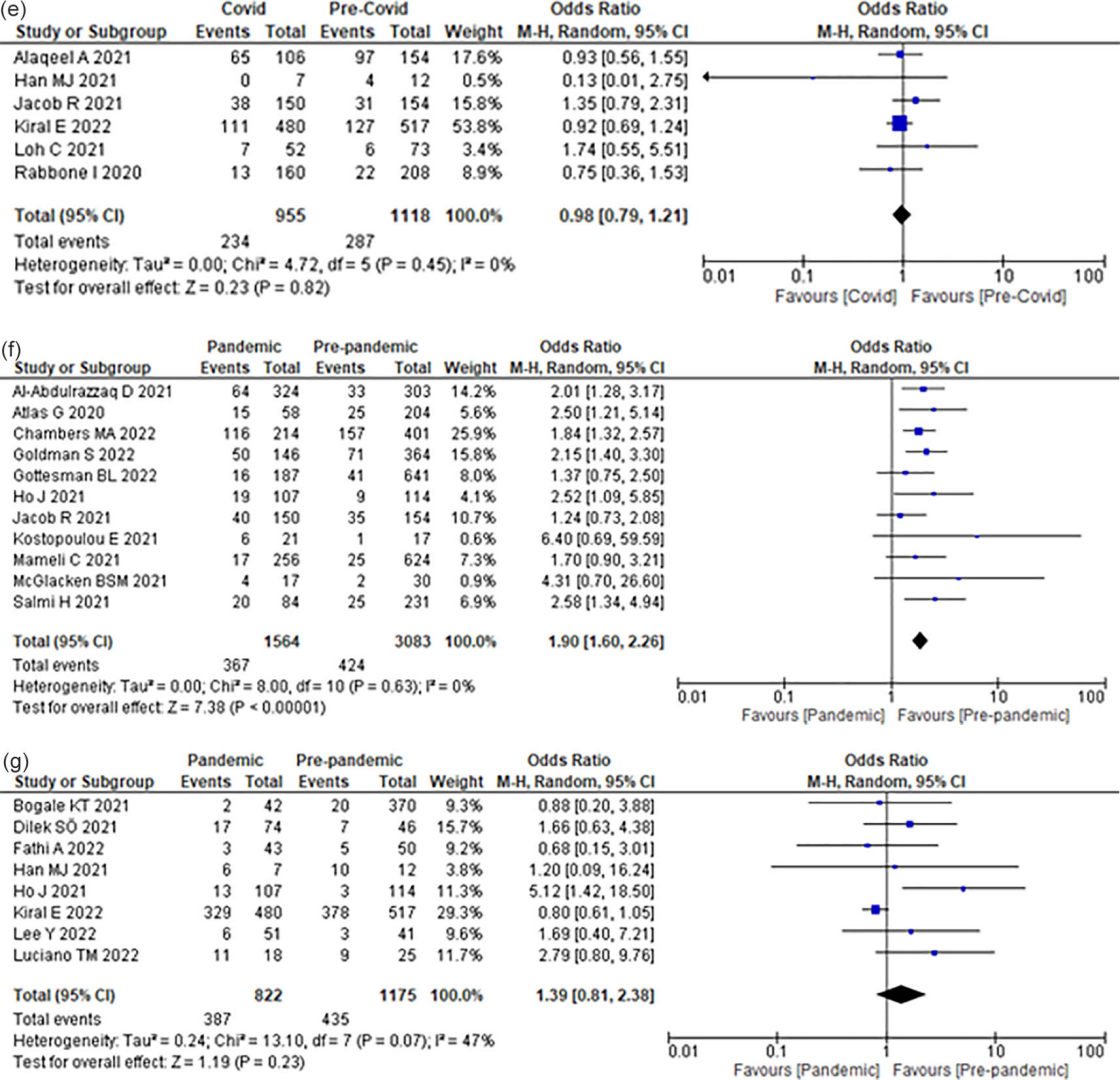
(a) Incidence of DKA in pediatric T1DM before and during the COVID-19 era according to the type of study design; (b) Incidence of DKA in pediatric T1DM before and during the COVID-19 era according to the continent of origin of the study; (c) Incidence of severe DKA in pediatric T1DM before and during the COVID-19 era; (d) Incidence of DKA in newly diagnosed pediatric T1DM before and during the COVID-19 era; (e) Incidence of DKA in established pediatric T1DM before and during the COVID-19 era; (f) Incidence of ICU admissions due to DKA in pediatric T1DM before and during the COVID-19 era; (g) Incidence of complications due to DKA in pediatric T1DM before and during the COVID-19 era.

Compared to the pre-COVID-19 era, the COVID-19 era increased the odds of DKA by 68% (OR 1.68; 95% CI 1.44–1.96) ( Figure 2a) among children with T1DM.

### Incidence of severe DKA

Compared to the pre-COVID-19 era, the COVID-19 era increased the odds of severe DKA by 84% (OR 1.84; 95% CI 1.59–2.12) ( Figure 2b) among children with T1DM.

### Incidence of severe DKA among newly diagnosed children with T1DM

Compared with the pre-COVID-19 era, the COVID-19 era increased the odds of DKA by 75% (OR 1.75; 95% CI 1.51–2.03) ( Figure 2c) among children with newly diagnosed T1DM.

### Incidence of severe DKA among children with established T1DM

Compared to the pre-COVID-19 era, the COVID-19 era did not significantly increase the odds of developing DKA (OR 0.98; 95% CI 0.79–1.21) ( Figure 2d) among children with established T1DM.

### Incidence of ICU admission due to DKA among T1DM pediatric patients

Compared with the pre-COVID-19 era, the COVID-19 era increased the odds of ICU admission due to DKA by 90% (OR 1.90; 95% CI 1.60–2.26) ( Figure 2e) among children with T1DM.

### Incidence of DKA complications among T1DM pediatric patients

Compared to the pre-COVID-19 era, the COVID-19 era did not significantly increase the odds of DKA complications (OR 1.39; 95% CI 0.81–2.38) ( Figure 2f) among T1DM pediatric patients. Authors reported different definitions for “DKA complications”, including acute kidney injury, ^46^ pulmonary edema, ^40^ stroke, ^58^ brain edema, ^40^^,^^43^ altered mental status, ^36^^,^^46^^,^^49^^,^^58^ treatment with hypertonic saline or mannitol, ^49^ hypokalemia, ^40^^,^^60^ hypocalcemia, or hypophosphatemia. ^40^ On the other hand, a study did not define “DKA complication.”

### Length of hospital stay among T1DM pediatric patients

Compared with the pre-COVID-19 era, the COVID-19 era did not significantly affect the duration of hospital stay due to DKA (MD 0.18; 95% CI -0.11–0.46) ( Figure 2g) among children with T1DM.

### Risk of mortality due to DKA

We found two studies ^37^^,^^43^ evaluating this outcome. Unfortunately, only one ^43^ of these two studies reported events during the pandemic. Consequently, we decided not to conduct a meta-analysis for this clinical outcome.

Of the 46 studies included, 40 had a low risk of bias and six had a high risk of bias according to assessment with the Newcastle-Ottawa Scale (NOS) tool ( Table 2).

**Table 2.** Bias assessment of the included studies according to NOS tool.

Author	Study design	Selection	Comparability	Outcome	Total	Conclusion
Al-Abdulrazzaq D. ^31^ 2021.	(MC) PCS.	****	**	***	9	Low risk
Alaqeel A. ^32^ 2021.	(MC) RCS.	***	**	***	8	Low risk
Alassaf A. ^33^ 2022.	(UC) RCS.	***	**	**	7	Low risk
Atlas G. ^34^ 2020.	(MC) RCS.	**	**	*	5	High risk
Boboc AA. ^35^ 2021.	(UC) RCS.	***	**	**	7	Low risk
Bogale KT. ^36^ 2021.	(UC) RCS.	***	**	***	8	Low risk
Chambers MA. ^37^ 2022.	(UC) RCS.	***	**	**	7	Low risk
Cherubini V. ^24^ 2022.	(MC) RCS.	***	**	**	7	Low risk
Danne T. ^39^ 2021.	(MC) CCS.	***	**	***	8	Low risk
Dilek SÖ. ^40^ 2021.	(UC) CSS.	***	**	**	7	Low risk
Donbaloğlu Z. ^41^ 2020.	(UC) CSS.	***	**	**	7	Low risk
Dżygało K. ^42^ 2020.	(UC) RCS.	***	**	***	8	Low risk
Fathi A. ^43^ 2022.	(UC) RCS.	***	**	**	7	Low risk
Goldman S. ^44^ 2022.	(MC) RCS.	**	**	*	5	High risk
Gottesman BL. ^45^ 2022.	(UC) CSS.	**	**	*	5	High risk
Han MJ. ^46^ 2021.	(MC) RCS.	***	**	**	7	Low risk
Hawkes CP. ^47^ 2021.	(UC) RCS.	**	**	*	5	High risk
Hernández HM. ^48^ 2022.	(MC) RCS.	***	**	***	8	Low risk
Ho J. ^49^ 2021.	(MC) RCS.	***	**	***	8	Low risk
Jacob R. ^50^ 2021.	(MC) CSS.	***	**	**	7	Low risk
Kamrath C. ^17^ 2020.	(MC) PCS.	****	**	***	9	Low risk
Kamrath C. ^51^ 2021.	(MC) PCS.	****	**	***	9	Low risk
Kaya G. ^52^ 2022.	(UC) RCS.	****	**	**	8	Low risk
Kiral E. ^53^ 2022.	(MC) RCS.	***	**	***	8	Low risk
Kostopoulou E. ^54^ 2021.	(MC) PCS.	****	**	***	9	Low risk
Lavik AR. ^55^ 2022.	(MC) RCS.	***	**	***	8	Low risk
Lawrence C. ^56^ 2021.	(UC) RCS.	***	**	***	8	Low risk
Lee MS. ^57^ 2022.	(UC) CSS.	***	**	**	7	Low risk
Lee Y. ^58^ 2022.	(MC) RCS.	***	**	***	8	Low risk
Loh C. ^59^ 2021.	(UC) CCS.	***	**	**	7	Low risk
Luciano TM. ^60^ 2022.	(UC) PCS.	****	**	***	9	Low risk
Mameli C. ^61^ 2021.	(MC) PCS.	****	**	***	9	Low risk
Marks BE. ^62^ 2021.	(UC) RCS.	***	**	***	8	Low risk
Mastromauro C. ^63^ 2022.	(UC) RCS.	***	**	***	8	Low risk
McGlacken BSM. ^64^ 2021.	(MC) CSS.	***	**	**	7	Low risk
Mönkemöller K. ^65^ 2021.	(MC) PCS.	****	**	***	9	Low risk
Nóvoa MY. ^66^ 2022.	(UC) RCS.	***	**	***	8	Low risk
Passanisi S. ^67^ 2022.	(MC) RCS.	***	**	***	8	Low risk
Rabbone I. ^68^ 2020.	(MC) PCS.	****	**	***	9	Low risk
Salmi H. ^69^ 2021.	(MC) RCS.	***	**	***	8	Low risk
Sellers EAC. ^70^ 2021.	(MC) RCS.	***	**	***	8	High risk
Tittel SR. ^71^ 2020.	(MC) RCS.	***	**	***	8	High risk
Vlad A. ^72^ 2021.	(MC) RCS.	***	**	***	8	Low risk
Vorgučin I. ^73^ 2022.	(MC) RCS.	***	**	***	8	Low risk
Wolf RM. ^74^ 2022.	(MC) RCS.	***	**	***	8	Low risk
Zubkiewicz A. ^75^ 2021.	(MC) RCS.	***	**	***	8	Low risk

PCS: prospective cohort study, RCS: retrospective cohort study, CCS: case-control study, NOS: Newcastle-Ottawa Scale tool.

Note: An asterisk (*) represents a star in each domain of the Newcastle–Ottawa scale (NOS) tool.

**Figure 3.**
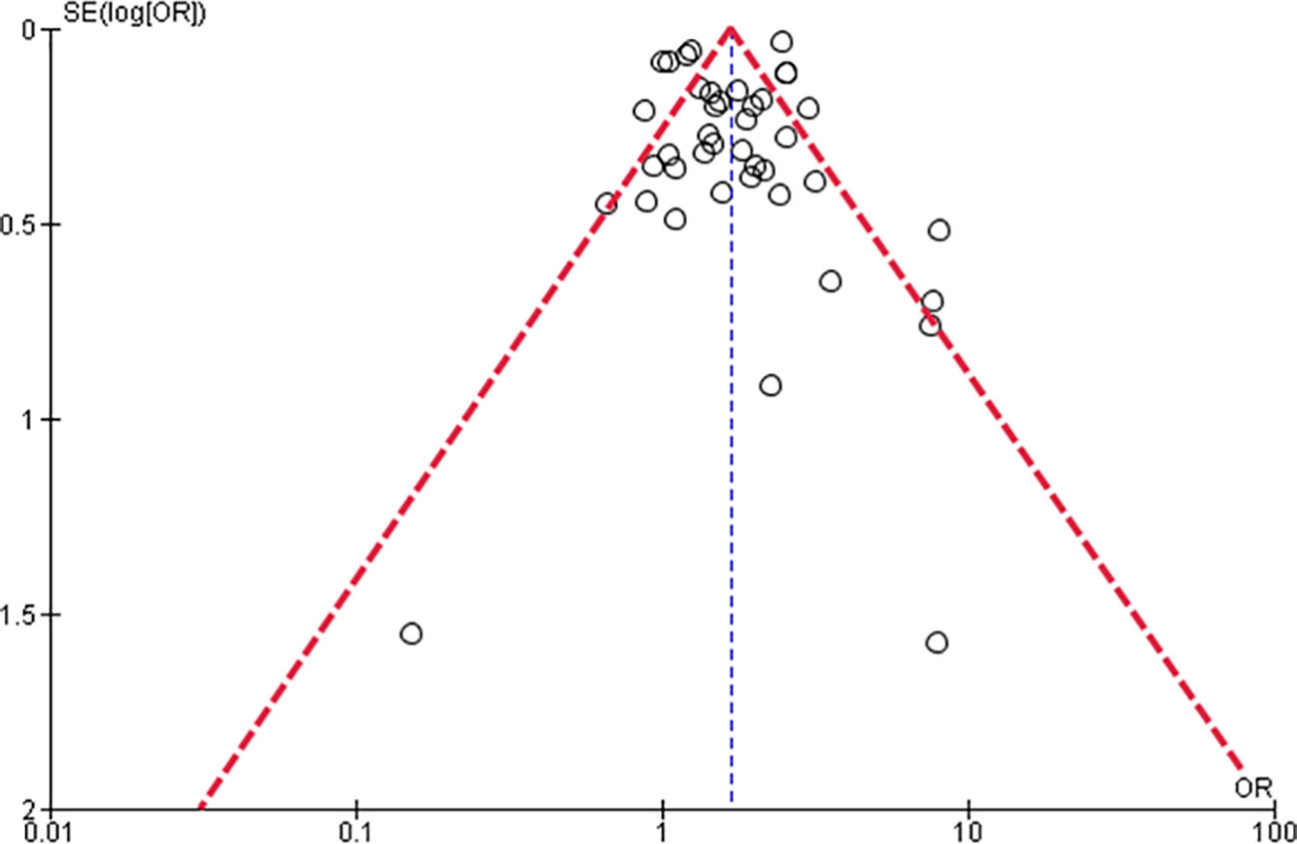
Funnel plot of the studies regarding the incidence of DKA in pediatric T1DM.

The funnel plot suggested publication bias ( Figure 3).

## Discussion

To our knowledge, this is the first systematic review and meta-analysis that asses nine outcomes associated with the effect of COVID-19 on pediatric T1DM and DKA. According to our results, the COVID-19 pandemic significantly increased the incidence of DKA (OR 1.68; 95% CI 1.44–1.96), severe DKA (OR 1.84; 95% CI 1.59–2.12), DKA in newly diagnosed T1DM (OR 1.75; 95% CI 1.51–2.03), and ICU admissions (OR 1.90; 95% CI 1.60–2.26). Conversely, we found no association between the COVID-19 pandemic and the incidence of T1DM, DKA in established T1DM, DKA complications, the length of hospitalization stay, and mortality ( Figure 2a-g). These findings are in agreement with other meta-analyses.

Nassar M *et al.* ^76^ conducted a systematic review to identify the prevalence, clinical presentation, and outcomes of T1DM in patients with COVID-19. They searched for observational studies in four databases. The results evaluated were the duration of hospital stay, general ward admission, ICU admission, frequency of DKA, serious hypoglycemia, and mortality. They included 15 papers in the qualitative analysis. They had reports that included information from both children and adults with COVID-19. The frequency of T1DM among patients with COVID-19 varied between 0 and 30%, while the prevalence of COVID-19 among patients with T1DM varied between 0 and 17%. The assessed outcomes ranged widely among the studies. Furthermore, the study’s duration of hospital stay, general ward admission, ICU admission, frequency of DKA, and serious hypoglycemia varied significantly among the included studies.

The systematic review by Nassar M *et al.* ^76^ has several limitations reported by the authors. First, they could not perform a meta-analysis because of the lack of studies with appropriate information. Besides, the characteristics of the participants differed widely among the studies. Therefore, in our meta-analysis, we excluded 13 of the 15 studies of the paper by Nassar M *et al.* because these studies combined adults with pediatric patients or did not have a control (pre-pandemic) group.

Rahmati M *et al.* ^12^ systematically explored the occurrence of *de novo* T1DM in children and its complications, such as diabetic ketoacidosis, previously and in the times of the pandemic of COVID-19. First, they carried out a systematic search of four databases. Then, they performed a quantitative synthesis comparing the probabilities of developing T1DM and diabetic ketoacidosis in children with T1DM before (the year 2019) and during (the year 2020) the pandemic. They also examined glycemic and glycated hemoglobin levels in pediatric participants with *de novo* T1DM previously and at the time of this pandemic. They found that the overall incidence ratio of T1DM in 2019 was 19.73 per 10 ^5^ children and 32.39 per 10 ^5^ in 2020. During 2020 the cases of *de novo* T1DM, diabetic ketoacidosis, and serious diabetic ketoacidosis raised significantly. Similarly, in 2020, the median glycemic and glycated hemoglobin levels in pediatric participants with *de novo* T1DM during the COVID-19 era increased notably. They concluded that the pandemic raised the likelihood of developing *de novo* T1DM, diabetic ketoacidosis, and serious diabetic ketoacidosis in children.

Rahmati M *et al.* ^12^ conducted heterogeneity and sensitivity analysis and assessed the possibility of publication bias. However, their paper only included studies covering the first wave of the SARS-CoV-2 pandemic. Conversely, our study includes more recent studies that covered subsequent waves. In addition, we excluded two studies of the review by Rahmati M *et al.*, one because it did not report DKA cases nor a numerator to calculate an incidence ratio, and the other because there was no control (pre-pandemic) group.

Alfayez OM *et al.* ^10^ conducted a systematic review aiming to study the characteristics of DKA before and during the pandemic of SARS-CoV-2 in children with T1DM. First, they searched for observational and found 20 documents on DKA. Then, they performed a random model analysis and reported that the pandemic, compared to the period before, significantly raised the probability of developing DKA and serious DKA. Similarly, pediatric patients with *de novo* T1DM presented a substantially greater risk of developing DKA during the pandemic than those patients during the pre-COVID-19 era. However, the heterogeneity was significant in all of these estimates (I ^2^ = 44%–71%). Two papers mentioned the likelihood of DKA among children with previously diagnosed T1DM, and this probability was not statistically increased during the COVID-19 era. The authors concluded that their research evidenced that DKA likelihood, particularly the chance of developing serious DKA, raised significantly during the COVID-19 period.

Alfayez OM *et al.* ^10^ reported subgroup and sensitivity analysis, and explored the possibility of publication bias. Although their systematic review collected fewer than half as many studies as ours, all included studies had an adequate control group. Consequently, their conclusions are closer to ours. Nevertheless, we highlight that of the studies included by these authors, one is a case-control study, ^39^ and four follow a cross-sectional design. ^40^^,^^50^^,^^57^^,^^64^ In studies of cross-sectional and case-control design, we only are able to assess odds, not risk. Therefore, a better measure of the effect size would have been to report odds ratios instead of risk ratios, ^77^^,^^78^ as the authors estimated.

Elgenidy A *et al.* ^11^ conducted a meta-analysis to investigate the increase of DKA in pediatrics at the time of the SARS-CoV-2 pandemic. In three databases, they looked for papers evaluating the frequency of diabetic ketoacidosis. The researchers reported 24 studies, including 124,597 pediatric patients with T1DM. Their main finding was that the pandemic raised statistically significantly the likelihood of developing DKA in children with *de novo* T1DM (RR 1.41; 95% CI 1.19–1.67; p < 0.01), particularly of those with serious DKA (RR 1.66: 95% CI 1.30–2.11) compared with the pre-COVID-19 era. Statistical heterogeneity was substantial (I ^2^ = 86% and 59%, respectively). They found no important rise in the probability of developing DKA during the pandemic in pre-existing T1DM or combined—de novo and pre-existing T1DM children compared with the pre-COVID-19 era. They concluded that the likelihood of DKA in children with *de novo* T1DM had risen in the time of the SARS-CoV-2 pandemic and tended to present in more serious forms.

Elgenidy A *et al.* performed subgroup and sensitivity analysis and evaluated the risk of publication bias. However, in our meta-analysis, we excluded 5 of the 17 studies quantitatively analyzed by Elgenidy A *et al.* because they did not report any of the events of interest or the lack of a control group. Moreover, these authors also included two case-control studies ^39^^,^^59^ and cross-sectional studies ^40^^,^^50^^,^^57^^,^^64^ and reported relative risks instead of odds ratios. Therefore, the same considerations previously mentioned for the meta-analysis of Alfayez OM *et al.* should apply.

The heterogeneity was significant in this systematic review and meta-analysis (I ^2^ > 90%, *p* < 0.05). According to the subgroup examination, the type of study design and the provenance region of the studies explained this lack of homogeneity among studies (test for subgroups difference I ^2^ = 83.2%, *p* = 0.003; I ^2^ > 49.4%, *p* = 0.11; respectively). Sensitivity analysis did not alter the global size estimate, showing good consistency. Because of the limited data among studies, we decided not to carry out subgroup analysis and meta-regression according to other variables. Unlike the study by Elgenidy A *et al.*, because most studies do not provide complete information, we did not perform subgroup according to the type of onset of diabetes ( *de novo*, established, or combined— *de novo* and established—T1DM) or the degree of diabetic ketoacidosis (serious, moderate, or mild). On the contrary, following Rahmati M *et al.* and Alfayez OM *et al.*, we analyzed the diabetes onset and the degree of DKA as an independent outcome.

We highlight several strengths in our meta-analysis: 1) the strategy search was comprehensive and compiled a more significant number of papers than any other previous systematic review or meta-analysis, 2) all the studies that we included involved a control (pre-pandemic) group, 3) all the papers that we included examined clinical—not surrogate—outcomes, and 4) we carried out sensitivity and subgroup analysis and examined for possible publication bias. Then, our conclusions are stronger than those previously reported by any other meta-analysis.

This study has important limitations: 1) heterogeneity was significant, 2) we were not able to carry out subgroup analyses regarding other essential factors such as age or sex, 3) it is possible that there exists a publication bias, as was suggested by our funnel plot, and finally, 4) we could not establish definite conclusions on other important outcomes such as the likelihood of T1DM, DKA complications, the duration of hospitalization stay, and mortality due to DKA. In addition, although we initially planned to perform subgroup analyzes according to sex, due to the scarcity of data (most studies combined information for both sexes), it was not possible to achieve this sub-analysis. In fact, none of the previously cited systematic reviews could perform a subgroup analysis according to sex.

## Conclusions

Our systematic review shows that the SARS-CoV-2 pandemic significantly impacted T1DM and DKA outcomes in pediatric patients. This pandemic increased 1) the risk of DKA, 2) the risk of serious DKA, 3) the risk of DKA in children with *de novo* T1DM, and 4) ICU admissions due to DKA. Conversely, the relation of the SARS-CoV-2 pandemic with other outcomes such as 1) the incidence of pediatric T1DM, 2) the incidence of DKA in established pediatric T1DM, 3) the incidence of complications due to DKA, 4) the length of hospitalization stay, and 5) the risk of mortality due to DKA, were not statistically significant. Nonetheless, clinicians should interpret these findings with caution due to several limitations. Consequently, more research is still necessary to improve knowledge of the relationship between SARS-CoV-2 and diabetic ketoacidosis. Nevertheless, our results imply that healthcare systems should be alert and prepared for a potential rise in diabetic ketoacidosis cases, especially severe DKA cases, in future waves of viral respiratory pandemics.

## Author roles

EDM-R: Conceptualization, Data Curation, Formal Analysis, Investigation, Methodology, Resources, Visualization, Writing, Original Draft Preparation; **FEL-J**: Data Curation, Formal Analysis, Investigation, Writing – Review & Editing; **BADT-H**: Investigation, Writing – Review & Editing; **GAV-T**: Conceptualization, Investigation, Supervision, Validation, Writing – Review & Editing

## Data Availability

Figshare: Figshare3: Raw data, https://doi.org/10.6084/m9.figshare.21644723. ^79^ This project contains the following underlying data:
- Figshare3-Raw data.xlsx (Search strategy, Table S1; Included primary studies, Table S2; Excluded primary studies) Figshare: Figshare4: *Extended data,* https://doi.org/10.6084/m9.figshare.21644786. ^80^ This project contains the following extended data:
- Figshare4-Supplementary materials.docx (Search strategy, Table S2- Excluded primary studies) Figshare: PRISMA checklist for “Impact of the Covid-19 pandemic on the incidence and clinical outcomes of diabetic ketoacidosis among children with type 1 diabetes: systematic review and meta-analysis”, https://doi.org/10.6084/m9.figshare.21625790. ^81^ Figshare: Figshare2: PRISMA Flowchart, https://doi.org/10.6084/m9.figshare.21625775. ^82^ Data are available under the terms of the Creative Commons Attribution 4.0 International license (CC-BY 4.0).
