# Supplementary material for: Impact of the COVID-19 pandemic on the incidence and clinical outcomes of diabetic ketoacidosis among male and female children with type 1 diabetes: systematic review and meta-analysis
Source: F1000Res. 2023 Aug 10;12:72. Originally published 2023 Jan 18. [Version 2] doi: 10.12688/f1000research.128687.2 (PMC11387936; doi:10.12688/f1000research.128687.2)
Supplement: Peer review history

# Reviewer report: Reviewer response for version 2

**DOI:** 10.5256/f1000research.154277.r243707

**Published:** 2024-09-10

**Authors:** Fatma Özgüç Çömlek

**Affiliations:** 1 Pediatric Endocrinology, Selçuk University Medical Facutly, Konya, Seçuklu, Turkey

**Title**

 The title is comprehensive and sufficient for the study. Perhaps it could be shortened to simply "among children" instead of " among male and female children."

**Abstract**

 “3) the incidence of KDA complications,” KDA-> DKA (KDA should write DKA)

**Introduction**

 “Another factor that could increase the incidence of T1DM, diabetic ketoacidosis (DKA), and severe DKA is the widespread use of steroids during the pandemic due to their ability to induce insulin resistance, hyperglycemia, and *de novo* DM. “

 In this article, we discuss Type 1 DM. There is no need to discuss the treatment protocols for COVID-19 that increase diabetes secondary to drugs. Also, our focus should be on children.

**Methods**

 A peer review whose field is statistics is the most appropriate situation.

**Discussion**

 The discussion and conclusion are sufficient and comprehensive.

Are the rationale for, and objectives of, the Systematic Review clearly stated?

Yes

Is the statistical analysis and its interpretation appropriate?

I cannot comment. A qualified statistician is required.

Are sufficient details of the methods and analysis provided to allow replication by others?

Yes

Are the conclusions drawn adequately supported by the results presented in the review?

Yes

Reviewer Expertise:

Pediatric Endocrinology

I confirm that I have read this submission and believe that I have an appropriate level of expertise to confirm that it is of an acceptable scientific standard.

# Reviewer report: Reviewer response for version 2

**DOI:** 10.5256/f1000research.154277.r243703

**Published:** 2024-02-19

**Authors:** Stefano Passanisi

**Affiliations:** 1 University of Messina, Messina, Italy

I have carefully reviewed the manuscript. The authors conducted a systematic review and meta-analysis to evaluate the incidence of new T1D cases, DKA incidence, severity, and associated outcomes during the COVID-19 pandemic. Despite the end of the pandemic, the topic remains relevant. The study is methodologically well-conducted, but some revisions are needed, particularly in language and the discussion section. Here are my specific comments:

- The first sentence regarding T1D definition should acknowledge viral infections as potential triggers for T1D onset.
- The description of the risk of bias assessment should be moved to the upper part of the results section, after presenting the final number of studies in the SR.
- In Table 1, consider using "non-specified" instead of "other" in the DKA criteria column.
- Figure 2, while separated into subgroups, is busy and may be challenging to read. Evaluate options for revision.
- Appreciate the analysis of T1D incidence based on different geographic areas in Figure 2b. Briefly describe this in the text.
- The discussion section needs expansion. Instead of describing other systematic reviews, delve into potential explanations for increasing T1D incidence and DKA, insights into the SARS-CoV-2 and T1D association, and the diabetogenic action of SARS-CoV-2. Refer to this recent literature review [1].
- A thorough revision by a native English speaker is recommended for text fluency.
- Address typos throughout the text for improved clarity (e.g. "Three reviewers (FEL-J, BADT-H, and GAV-T) independently. Three independent reviewers (FEL-J, BADT-H, and GAV-T) examined ...."

Are the rationale for, and objectives of, the Systematic Review clearly stated?

Yes

Is the statistical analysis and its interpretation appropriate?

Yes

Are sufficient details of the methods and analysis provided to allow replication by others?

Yes

Are the conclusions drawn adequately supported by the results presented in the review?

Partly

Reviewer Expertise:

Pediatrics, diabetes, allergy

I confirm that I have read this submission and believe that I have an appropriate level of expertise to confirm that it is of an acceptable scientific standard, however I have significant reservations, as outlined above.

## References

[1] : Examining the associations between COVID-19 infection and pediatric type 1 diabetes. *Expert Rev Clin Immunol* .2023;19(5) : 10.1080/1744666X.2023.2189587 489-497 10.1080/1744666X.2023.2189587 36888906

# Reviewer report: Reviewer response for version 1

**DOI:** 10.5256/f1000research.141300.r172554

**Published:** 2023-07-25

**Authors:** Carlos J. Toro-Huamanchumo

**Affiliations:** 1 Unidad de Investigación para la Generación y Síntesis de Evidencias en Salud, Universidad San Ignacio de Loyola, Lima District, Lima Region, Peru

I have carefully gone through your manuscript titled "Impact of the COVID-19 pandemic on the incidence and clinical outcomes of diabetic ketoacidosis among male and female children with type 1 diabetes: systematic review and meta-analysis". Below are my comments and suggestions for improving your submission:

1. The objective of evaluating the incidence of T1D is not clearly stated, especially since the title mentions that your population is children with T1D. It would seem this is not a variable as all subjects already have this diagnosis. Could you clarify this further?
2. It is not clear how clinical trials were considered as potential studies to be selected. Could you elaborate on the criteria used for this selection?
3. When you state "We excluded case reports, case series, duplicated publications, and papers in which most patients were >18 years old or had other types of diabetes mellitus", could you specify what you mean by "most"? Does this refer to >50%? >75%? This point needs to be explicitly clarified.
4. The process of how four independent reviewers examined the articles and a fifth investigator resolved discrepancies is unclear, especially given that there are only four authors listed in total. Please note that only three authors are listed on PROSPERO.
5. The eligibility criteria need to be more precisely defined. The PECO question seems more oriented towards assessing whether having COVID-19 is associated with any of those outcomes, and not whether the incidence of each of the outcomes increased during the pandemic vs. pre-pandemic. In this sense, rather than formulating a PECO, it may be more useful to describe the inclusion criteria for studies in detail (as some previous systematic reviews have done).
6. Please clarify why you preferred to use OR instead of RR.
7. The statistical procedure for obtaining MD for the duration of hospital stay is not mentioned. Could you provide more detail on this?
8. I strongly recommend presenting the complete results of the NOS, not just the final outcome.

Are the rationale for, and objectives of, the Systematic Review clearly stated?

Partly

Is the statistical analysis and its interpretation appropriate?

Partly

Are sufficient details of the methods and analysis provided to allow replication by others?

Partly

Are the conclusions drawn adequately supported by the results presented in the review?

Partly

Reviewer Expertise:

Evidence-Based Medicine, Epidemiology & Public Health, Obesity & Metabolism

I confirm that I have read this submission and believe that I have an appropriate level of expertise to confirm that it is of an acceptable scientific standard, however I have significant reservations, as outlined above.

# Response

**Published:** 2023-08-06

**Authors:** Edinson Dante Meregildo-Rodriguez

**Affiliations:** Hospital Regional Lambayeque, Peru

The authors appreciate the reviewer's comments, which will enhance this article's quality and methodological rigor.

**The objective of evaluating the incidence of T1D is not clearly stated, especially since the title mentions that your population is children with T1D. It would seem this is not a variable as all subjects already have this diagnosis. Could you clarify this further?**

**Replay:** Thank you for this valuable comment. As we stated in the last paragraph of the Introduction, we aimed to determine the impact of this pandemic on the risk (probability) of developing pediatric T1DM, DKA, severe DKA, DKA in newly diagnosed and established T1DM, etc. So, we collected studies with participants, children with and without T1DM, DKA, severe DKA, DKA in newly diagnosed and established T1DM, etc. However, we must recognize that our PECO strategy did not fully reflect this intention. Therefore, in this new version of the manuscript, we have slightly modified the structure of the PECO question, and we have described with more detail the inclusion criteria following the example of previous similar systematic reviews.

**It is not clear how clinical trials were considered as potential studies to be selected. Could you elaborate on the criteria used for this selection?**

**Replay:** Thank you for this insightful observation. We included RCTs in our strategy search to carry out a broad and sensitive search. However, from the methodological and ethical point of view, and considering the exposure was "COVID-19" OR "SARS-CoV-2", it was improbable to find any published RCT, which was confirmed by our results. All the studies were observational.

**When you state "We excluded case reports, case series, duplicated publications, and papers in which most patients were >18 years old or had other types of diabetes mellitus", could you specify what you mean by "most"? Does this refer to >50%? >75%? This point needs to be explicitly clarified.**

**Replay:** Thank you for this comment. According to The Cochrane Handbook, the criteria for considering the population included in studies in a systematic review should be sufficiently broad to encompass the likely diversity of studies and the likely scenarios in which the interventions will be used, but narrow enough to ensure that a meaningful answer can be obtained when studies are considered together. A cutoff of more than 50% ensures that most participants in the included studies are children, which increases the likelihood that the results are relevant to children. Then, In the new version of the manuscript, we have included a small paragraph indicating that we considered a >50% cutoff for excluding studies.

**The process of how four independent reviewers examined the articles and a fifth investigator resolved discrepancies is unclear, especially given that there are only four authors listed in total. Please note that only three authors are listed on PROSPERO.**

**Replay:** Thank you for this insightful comment. You are right. Consequently, we have made the pertinent corrections in this revised manuscript version. Furthermore, we have updated the protocol in PROSPERO, including the four authors.

**The eligibility criteria need to be more precisely defined. The PECO question seems more oriented towards assessing whether having COVID-19 is associated with any of those outcomes, and not whether the incidence of each of the outcomes increased during the pandemic vs. pre-pandemic. In this sense, rather than formulating a PECO, it may be more useful to describe the inclusion criteria for studies in detail (as some previous systematic reviews have done).**

**Replay:** Thank you for this accurate comment. We have made the following modifications in our manuscript: 1) we have modified the Population component of our PECO question, and 2) described with more detail the inclusion criteria following the example of previous similar systematic reviews.

**Please clarify why you preferred to use OR instead of RR.**

**Replay:** Thank you for this insightful comment. Of the 46 studies included in this review, six studies were cross-sectional studies (CSS), two papers were case-control studies (CCS), and thirty-eight documents were prospective or retrospective cohort studies (PCS, RCS). The only measure of association that these study designs have is the OR. Consequently, we used OR, not RR.

**The statistical procedure for obtaining MD for the duration of hospital stay is not mentioned. Could you provide more detail on this?**

**Replay:** Thank you for this valuable comment. In this revised version of our manuscript, we have modified the respective paragraph detailing the statistical method used for obtaining MD and SD. Furthermore, we have included the corresponding references that support these statements.

**I strongly recommend presenting the complete results of the NOS, not just the final outcome.**

**Replay:** Thank you for this important. In this revised version of our manuscript, we have modified "Table 2. Bias assessment of the included studies," presenting the complete Newcastle-Ottawa Scale (NOS) tool.
